# Combinational blockade of MET and PD-L1 improves pancreatic cancer immunotherapeutic efficacy

**DOI:** 10.1186/s13046-021-02055-w

**Published:** 2021-09-03

**Authors:** Enliang Li, Xing Huang, Gang Zhang, Tingbo Liang

**Affiliations:** 1 Zhejiang Provincial Key Laboratory of Pancreatic Disease, the First Affiliated Hospital, School of Medicine, Zhejiang University, 310009 Hangzhou, Zhejiang China; 2 Department of Hepatobiliary and Pancreatic Surgery, the First Affiliated Hospital, School of Medicine, Zhejiang University, 310003 Hangzhou, Zhejiang China; 3 Innovation Center for the Study of Pancreatic Diseases, 310009 Hangzhou, Zhejiang China; 4 Zhejiang Clinical Research Center of Hepatobiliary and Pancreatic Disease, 310003 Hangzhou, Zhejiang China; 5 Zhejiang University Cancer Center, 310058 Hangzhou, Zhejiang China; 6 Research Center for Healthcare Data Science, Zhejiang Lab, 310003 Hangzhou, Zhejiang China

**Keywords:** Pancreatic cancer, Receptor tyrosine kinase, MET, Immune checkpoint, PD-L1, Targeted and combined immunotherapy

## Abstract

**Background:**

Dysregulated expression and activation of receptor tyrosine kinases (RTKs) are associated with a range of human cancers. However, current RTK-targeting strategies exert little effect on pancreatic cancer, a highly malignant tumor with complex immune microenvironment. Given that immunotherapy for pancreatic cancer still remains challenging, this study aimed to elucidate the prognostic role of RTKs in pancreatic tumors with different immunological backgrounds and investigate their targeting potential in pancreatic cancer immunotherapy.

**Methods:**

Kaplan–Meier plotter was used to analyze the prognostic significance of each of the all-known RTKs to date in immune “hot” and “cold” pancreatic cancers. Gene Expression Profiling Interactive Analysis-2 was applied to assess the differential expression of RTKs between pancreatic tumors and normal pancreatic tissues, as well as its correlation with immune checkpoints (ICPs). One hundred and fifty in-house clinical tissue specimens of pancreatic cancer were collected for expression and correlation validation via immunohistochemical analysis. Two pancreatic cancer cell lines were used to demonstrate the regulatory effects of RTKs on ICPs by biochemistry and flow cytometry. Two *in vivo* models bearing pancreatic tumors were jointly applied to investigate the combinational regimen of RTK inhibition and immune checkpoint blockade for pancreatic cancer immunotherapy.

**Results:**

MET was identified as a pancreatic cancer-specific RTK, which is significantly associated with prognosis in both immune “hot” and “cold” pancreatic cancers. MET was observed to be highly upregulated in pancreatic cancer tissues, and positively correlated with PD-L1 levels. Elevated MET and PD-L1 expressions were closely associated with lymph node metastasis, tumor TNM stage, and overall survival in pancreatic cancer. Mechanistically, MET could interact with PD-L1, and maintain its expression level in multiple ways. MET deficiency was found to facilitate lymphocyte infiltration into pancreatic tumors. Finally, significant benefits of combining MET inhibition with PD-1/PD-L1 blockage were verified in both orthotopic and subcutaneous mouse models of pancreatic cancer.

**Conclusions:**

This study systematically investigated the potential effectiveness of a novel pancreatic cancer immunotherapy targeting RTKs, and revealed the function of MET in PD-L1 regulation as well as the combined therapeutic efficacy of MET and PD-L1 in pancreatic cancer.

**Supplementary Information:**

The online version contains supplementary material available at 10.1186/s13046-021-02055-w.

## Background

As an aggressive malignant cancer driven by multiple molecular mechanisms, pancreatic cancer is associated with an extremely high mortality rate [1–3]. Pancreatic ductal adenocarcinoma (PDAC), which is the most common type of pancreatic cancer, has become the fourth deadliest malignancy in Western countries [4, 5]. Further, given the late diagnosis, rapid metastatic progression, resistance to conventional therapeutics, and increased incidence rate of pancreatic cancer, it is expected to become the second leading cause of cancer-related deaths in the United States by 2030 [6]. This poses a grave threat to human health and survival. Thus, it is imperative to identify more efficacious therapies against this dreadful disease, especially because of its intrinsic resistance to the immune system [7–9].

Receptor tyrosine kinases (RTKs) are high-affinity cell surface receptors that mediate environmental signal transmission and cellular responses [10–13]. Dysregulated expression and aberrant activation of RTKs are associated with a range of human disease-related processes, especially tumorigenesis and cancer progression [14, 15]. Accordingly, multiple RTK-targeting drugs have been developed and applied, benefiting numerous cancer patients [16]. However, current RTK-targeting therapies unfortunately exert little effect on pancreatic cancer. Epidermal growth factor receptor (EGFR) blockage in PDAC previously reported with disappointing outcomes and dampened the enthusiasm in the examination of RTKs for pancreatic cancer therapy [17]. Moreover, the association between RTKs and the immune status in pancreatic cancer still remains unclear. Accordingly, to enhance our understanding of the efficacy of RTK-targeting therapy, further investigation with regard to the selection of targets and therapeutic strategies is warranted.

MET is a classical RTK and the receptor of hepatocyte growth factor (HGF) [18–20]. MET was observed to be upregulated in pancreatic cancer, and that was associated with tumor grade [21]. Further, the HGF-MET axis is involved in the metastatic progression of pancreatic cancer and functions as a bridge linking the tumor with stroma, resulting in a positive loop for pancreatic cancer progression [21]. In view of these findings, there is continuous research on targeting HGF-MET signaling in pancreatic cancer with MET overexpression. However, few therapeutic outcomes have been obtained from these studies because of HGF-MET-targeting drug resistance in pancreatic cancer [18, 22]. Recent reports fortunately provide novel directions for pancreatic cancer treatment. Noguchi et al. reported that the upregulation of MET induces gemcitabine resistance in mice with pancreatic cancer[23]. Tomihara et al. showed that radiation therapy upregulates MET and activates its downstream signaling in PDAC [24]. Rucki et al. suggested that treatment strategies targeting HGF/MET and Hedgehog pathways alleviate monodrug resistance; they further reported that HGF/MET-targeted drug therapy in combination with chemotherapy shows more significant anti-tumor effects compared with monotherapy [25]. These data indicate that combination therapy enhances the therapeutic efficacy of MET-targeted drugs and may serve as a new treatment strategy for pancreatic cancer.

The property of the immune system to discriminate tumor-derived antigens as non-self and to be activated to eliminate cancer cells indicates a connection of tumor immunotherapy with MET-targeted therapy [19, 26]. Immune checkpoint (ICP) inhibitors targeting programmed cell death protein 1 (PD-1)/PD-1 ligand 1 (PD-L1) are currently the most popular weapon in cancer immunotherapy. PD-L1 binds to PD-1 to inhibit T cell activation and cytokine production, resulting in cancer cell immune tolerance and survival [27, 28]. PD-L1-targeted drugs block this interaction and reactivate the immune system to attack and eradicate tumor cells in cancer patients with PD-L1 overexpression; this has benefited a proportion of patients in multiple cancers [29, 30]. However, the therapeutic efficacy of PD-L1 blockage remains limited in pancreatic cancer [31]. In clinical trials, the administration of ipilimumab and BMS-936,559 (an anti-PD-L1 monoclonal antibody) showed poor anti-tumor effects in advanced pancreatic cancer cases [32–34]. However, an increasing number of preclinical studies have shown that combination therapy effectively improves the efficacy of PD-L1 blockage. For example, Pan et al. reported that JQ1 and anti-PD-L1 antibody synergistically inhibited PDAC [35]. Mace et al. showed that joint blockage of IL-6 and PD-L1 suppresses pancreatic cancer growth in mice and elevates the levels of intra-tumoral effector T cells [36]. Therefore, exploring the regulatory mechanisms associated with PD-L1 expression could help identify biomarkers for predicting the therapeutic response after ICP blockage (ICB) in PDAC.

In this study, we systematically investigated the potential effectiveness of targeting RTKs as a novel strategy for pancreatic cancer treatment. We first focused on elucidating the prognostic role of all known RTKs in pancreatic tumors with different immunological backgrounds. Furthermore, we identified pancreatic cancer-specific RTKs, which are significantly upregulated in pancreatic tumors and associated with ICPs. Most importantly, we determined the endogenous correlation of MET with PD-L1 and the function of MET in the regulation of PD-L1 expression as well as the efficacy of their combination therapy in pancreatic cancer treatment. Thus, our study provides novel insights for the selection of targets and treatment strategies for pancreatic cancer patients.

## Methods

### Data collection

To analyze prognoses and expression levels of RTKs as well as to determine their correlation with ICPs in pancreatic cancer, the Pancreatic Adenocarcinoma datasets from TCGA database (http://cancergenome.nih.gov) were collected by two individual web servers.

### Kaplan–Meier survival curve analysis

The Kaplan–Meier plotter (KM-plotter, http://kmplot.com, updated on 2020.07.22) [37] was used to analyze the effects of each RTK on the prognosis of immune “hot” and “cold” pancreatic cancers. The KM-plotter can assess the effect of 54k genes (mRNA, miRNA, and proteins) on survival in 21 cancer types. The database sources include GEO, EGA, and TCGA. The primary purpose of this tool is meta-analysis-based discovery and validation of survival biomarkers. In this study, KM-plotter was used to analyze the prognostic roles of each RTK in both immune “hot” (CD8 + T-cell enriched) and “cold” (CD8 + T-cell decreased) pancreatic cancers. Overall survival analysis of each RTK was performed using the Kaplan–Meier method with the best cutoff to divide patients into lower and higher expression groups. The log-rank test (Mantel–Cox test) was used for hypothesis testing. The cox proportional hazards regression model was applied to calculate the hazard ratio, and a *p*-value < 0.05 was considered to be statistically significant.

### Gene expression profiling interactive analysis

Gene Expression Profiling Interactive Analysis (GEPIA, http://gepia2.cancer-pku.cn, version 2) [38] is an open-access online tool for the interactive exploration of RNA sequencing expression data of 9,736 tumors and 8,587 normal samples from the TCGA and GTEx projects. GEPIA uses the UCSC Xena project-based datasets (http://xena.ucsc.edu), following a standard processing pipeline to avoid data imbalance[39, 40]. In this study, GEPIA was used to analyze the differential expressions of RTKs between pancreatic cancer tissues and normal pancreatic tissues, as well as the correlations of RTKs with ICPs in pancreatic cancer. One-way ANOVA was used to analyze the differential expression of RTKs, and genes with |log2FC| values > 1 and q values < 0.05 were considered to be differentially expressed. For correlation analyses, we focused on the RTKs that were upregulated in pancreatic cancer tissues compared with normal pancreatic tissues, and six most well-established inhibitory ICPs on the tumor side were selected and integrated as the ICP signature in our study; these included PD-L1 (also known as CD274 and B7H1), CD276 (also known as B7H3), CD155 (also known as PVR), CD112 (also known as NECTIN2 and PVRL2), LGALS9 (also known as Galectin 9), and HVEM (also known as CD258 and LIGHT). Spearman correlation analysis was used to analyze the pair-wise gene expression correlations between RTKs and ICP signature, and a *p*-value < 0.05 was considered to indicate a statistically significant difference.

### Human PDAC specimen collection

Overall, 140 human PDAC tumor tissue specimens for tumor tissue microarray as well as 10 human PDAC-paired tumor and adjacent noncancerous tissue samples were collected from the Department of Hepatobiliary and Pancreatic Surgery, the First Affiliated Hospital of Zhejiang University School of Medicine. All individuals underwent surgery for primary PDAC, with no previous history of chemotherapy or radiation therapy. Written informed consent was provided by all patients in this trial, and the study protocol was approved by the medical ethics committee of our hospital.

### Cell culture

Human PDAC cell line BXPC-3 was provided by the American Type Culture Collection (ATCC, USA). Mouse PDAC KPC cells (from a spontaneous tumor in an LSL-Kras G12D/+, LSL-Trp53 R172H/+ Pdx1-Cre mouse) were kindly provided by Prof. Raghu Kalluri’s laboratory (Department of Cancer Biology, Division of Science, MD Anderson Cancer Center, Houston, TX, USA). BXPC-3 and KPC cells were cultured in RPMI 1640 (Thermo Fisher, USA) containing 10 % fetal bovine serum (FBS, Thermo Fisher, USA) and 1 % penicillin and streptomycin (Genom, China) at 37 °C with 5 % CO_2_.

### Cell transfection and treatment

Nonsense and MET shRNA plasmids manufactured by Shanghai OBiO (China) were used to stably silence MET expression in mouse and human PDAC cell lines. The targeting sequences were described as follows: Human-MET, shRNA-1: GCATGTCA ACATCGCTCTA and shRNA-2: GCTGGTGTTGTCTCAATAT, and Mouse-MET, shRNA-1: CCAAAGTTCTGCTTGGCAA and shRNA-2: GCAGCCTGATTGTGCATTT. CMTM6 (NCBI Ref Seq: NM_017801.2) overexpression plasmids (HG11340-NF, Sino Biological), IFNγ (100 ng/mL, PeproTech), MG-132 (5 μm/mL, Selleck), and cycloheximide (100 µg/mL, Selleck) were administered to explore the regulatory mechanism of MET on PD-L1.

### Quantitative real-time PCR (qPCR)

qPCR was conducted based on a previous report [41]. Total RNA was isolated using TRIzol reagent (Invitrogen, USA). Following this, cDNA was synthesized using PrimeScript RT reagent Kit (Takara). SYBR Green Real-Time PCR Master Mix (Takara) was used for qPCR on an Applied QuantStudio™ 5 System, as directed by the manufacturer. The 2^−ΔΔCt^ method was followed for the analysis. Primers for human c-MET (Cat HP100082) and PD-L1 (Cat HP100170) were provided by Sino Biological (Beijing, China).

### Western blotting

Immunoblotting was performed based on a previous report [41] and has been briefly described below. Total protein for Western blotting was obtained using radioimmunoprecipitation assay (RIPA) lysis buffer supplemented with protease inhibitor and phosphorylase inhibitors, separated via sodium dodecyl sulfate polyacrylamide gel electrophoresis (SDS-PAGE), and electro-transferred onto PVDF membranes (Millipore, USA). The following primary antibodies were used for protein detection: anti-MET (1:1000, #8198; Cell Signaling Technology), anti-MET (1:1000, #25869-1-AP, Proteintech), anti-STAT1 (1:1000, #14,994, Cell Signaling Technology), anti-pSTAT1 Tyr701 (1:1000, #7649, Cell Signaling Technology), anti-PD-L1 (1:1000, #13,684,Cell Signaling Technology), anti-PD-L1 (1:1000, 66248-1-Ig, Proteintech), anti-GAPDH (1:1000, AF1186, Beyotime), and anti-β-Actin (1:1000, #AF5003, Beyotime). Secondary HRP-conjugated goat anti-mouse IgG (1:50,000, A0216, Beyotime) or anti-rabbit IgG (1:50,000, A0208, Beyotime) were also used. The Clinx ChemiScope series System (Clinx Science Instrument, China) was used for visualization.

### Immunohistochemistry (IHC)

IHC assays were performed to assess the expressions of MET and PD-L1. Ten paired paraffin-embedded PDAC patients’ tumor tissues and their adjacent normal pancreatic tissues were cut into 4-µm-thick slices, and tissue microarrays of 140 PDAC samples were individually subjected to IHC analysis. After antigen recovery, these were blocked with goat serum for 30 min at ambient temperature, followed by incubation with the anti-MET antibody (1:100, #25869-1-AP, Proteintech), anti-PD-L1 antibodies (1:10,000, #66248-1-Ig, Proteintech), and anti-CD11B antibodies (1:50,000, #ab133357, Abcam) overnight at 4 °C, and corresponding biotinylated secondary antibodies for 2 h at ambient temperature. DAB kits were used for detection, and counterstaining was performed using hematoxylin and eosin before analysis under a microscope (Leica, Germany). The immunostained tissues were reviewed and graded by two independent pathologists. The samples were scored using the histochemistry score (H-score) method combining the values of immunoreaction intensity and percentage of tumor-cell staining.

### Co-immunoprecipitation

Co-immunoprecipitation experiments were performed using Protein A/G Magnetic Beads (Bimake, B23201) according to the manufacturer’s instructions. In brief, cells were washed three times with ice-cold PBS and lysed with IP Cell Lysis Buffer on ice with gentle periodic mixing for 30 min. Cell lysates were incubated with magnetic beads-antibody complexes (5 µg antibody and 25 µL protein A/G magnetic beads) overnight at 4 °C. After washing with the elution buffer, the protein complexes were boiled and subjected to Western blotting. The protein complexes were washed three times with elution buffer, followed by resuspension in the loading buffer and heating (95 °C for 5 min). The immunoprecipitates were assessed by immunoblotting. The following antibodies were used for protein detection: anti-MET (1:50, cell signaling technology, 8198), anti-PD-L1 (1:50, cell signaling technology, 13,684), and anti-CMTM6 (1:100, cell signaling technology, 19,130).

### Flow cytometry

Tumor tissue samples were washed with 3× PBS, minced, and treated with digestive solution composing of 95 % RPMI 1640, 2 % FBS, 1 % collagenase IV solution, 1 % DNase I, and 1 % Dispase II at 37 °C. Following this, single cells obtained by centrifugation (200 rpm for 1 h) were filtered using a 70-µm nylon mesh (Corning) and stained with anti-mouse CD274, CD45, CD3, CD8, CD4, Granzyme B (GZMB), IFNγ, TNF-α, mouse Fc block (2.4G2), anti-human CD274, and human Fc block (BD Biosciences or BioLegend) separately, as directed by the corresponding manufacturers. A Beckman FACS flow cytometer was used for data analysis.

### Mouse models

Male C57BL/6 mice (6 weeks) provided by Shanghai Experimental Animal Center (Shanghai, China) were housed under Specific-pathogen-free (SPF) conditions. All animal protocols in this study were approved by the Animal Care and Use Committee of the Zhejiang University School of Medicine. In xenograft studies, 5 × 10^5^ KPC cells were subcutaneously or orthotopically injected. Following this, xenograft tumors were assessed at three-day intervals. When the tumor volume reached approximately 30 mm^3^, the mice were intraperitoneally injected with PD-L1 antibody (200 µg, Bio X Cell) or PD-1 antibody (100 µg, Bio X Cell) once every three days or orally administered capmatinib daily (with 0.5 % methylcellulose and 5 % dimethyl acetamide) at a dose of 10 mg/kg. The tumor volume was determined as follows: (length × width^2^)/2. After two weeks of treatment, the mice underwent CO_2_ inhalation for euthanasia, and tumor specimens were collected.

### Statistical analyses

SPSS v22 (SPSS, USA) and GraphPad Prism 7 (GraphPad Software, USA) were used for data analysis. Data are presented as mean ± standard deviation (SD). Continuous and categorical variables were compared by unpaired Student’s t-test and the Chi-squared or Fisher exact test, respectively. Overall survival was compared by the Kaplan–Meier method, and significance was determined by log-rank test. *P* < 0.05 was considered statistically significant.

## Results

### RTKs are prognostic factors for immune “hot” pancreatic cancer

A total of 58 RTKs have been identified to date [10, 42]. RTKs can be categorized into 20 types based on structural homology, with the following categories in the respective types: (I) EGFR and ERBB2-4; (II) INSR, IGF1R, and INSRR; (III) PDGFRA, PDGFRB, CSF1R, KIT, and FLT3; (IV) FLT1, KDR, and FLT4; (V) FGFR1-4; (VI) PTK7; (VII) NTRK1-3; (VIII) ROR1-2; (IX) MUSK; (X) MET and MST1R; (XI) TYRO3, AXL, and MERTK; (XII) TIE1 and TEK; (XIII) EPHA1-8, EPHA10, EPHB1-4, and EPHB6; (XIV) RET; (XV) RYK; (XVI) DDR1-2; (XVII) ROS1; (XVIII) AATK and LMTK2-3; (XIX) LTK and ALK; and (XX) STYK1 (Table 1).

**Table 1:** Prognostic profile of RTK family subtypes in pancreatic cancer

Type	RTK	CD8 + T-cells-enriched			CD8 + T-cells-decreased		
		Prognosis	*P*-value	HR	Prognosis	*P*-value	HR
I	EGFR	Unfavor	0.00046	5.94	Unfavor	0.061	1.67
	ERBB2	Unfavor	0.0015	5.57	Unfavor	0.077	1.59
	ERBB3	Unfavor	0.0097	2.54	Unfavor	0.054	1.71
	ERBB4	Unfavor	0.054	1.96	Favor	0.13	0.66
II	INSR	Favor	0.088	0.48	Unfavor	0.31	1.32
	IGF1R	Unfavor	0.11	1.74	Favor	0.12	0.67
	INSRR	Unfavor	0.24	1.65	Favor	0.03	0.49
III	PDGFRA	Unfavor	0.043	2.25	Unfavor	0.093	1.75
	PDGFRB	Unfavor	0.018	2.29	Unfavor	0.33	1.3
	CSF1R	Unfavor	0.019	2.31	Favor	0.17	0.69
	KIT	Unfavor	0.18	1.77	Favor	0.21	0.7
	FLT3	Unfavor	0.24	1.58	Unfavor	0.34	1.29
IV	FLT1	Favor	0.04	0.47	Unfavor	0.041	1.72
	KDR	Favor	0.12	0.53	Favor	0.22	0.71
	FLT4	Favor	0.048	0.5	Favor	0.31	0.76
V	FGFR1	Favor	0.0031	0.35	Favor	0.44	0.81
	FGFR2	Unfavor	0.097	2.22	Favor	0.02	0.48
	FGFR3	Favor	0.08	0.44	Unfavor	0.2	1.44
	FGFR4	Favor	0.32	0.67	Favor	0.14	0.65
VI	PTK7	Unfavor	0.0045	2.66	Unfavor	0.15	1.47
VII	NTRK1	Unfavor	0.24	1.59	Favor	0.31	0.71
	NTRK2	Unfavor	0.49	1.29	Favor	0.15	0.65
	NTRK3	Unfavor	0.17	1.78	Favor	0.48	0.82
VIII	ROR1	Unfavor	0.0054	2.64	Unfavor	0.12	1.54
	ROR2	Unfavor	0.11	1.76	Favor	0.01	0.49
IX	MUSK	Unfavor	0.026	3.11	Unfavor	0.34	1.28
X	MET	Unfavor	0.0014	5.64	Unfavor	1.4e-05	3.01
	MST1R	Unfavor	0.0093	2.48	Unfavor	0.084	1.74
XI	TYRO3	Unfavor	0.17	1.65	Favor	0.14	0.67
	AXL	Unfavor	0.0044	4.27	Unfavor	0.064	1.63
	MERTK	Unfavor	0.06	2.15	Favor	0.08	0.63
XII	TIE1	Favor	0.089	0.55	Favor	0.086	0.64
	TEK	Unfavor	0.37	1.47	Favor	0.18	0.7
XIII	EPHA1	Unfavor	0.24	1.54	Favor	0.25	0.74
	EPHA2	Unfavor	0.021	2.24	Unfavor	0.073	1.75
	EPHA3	Unfavor	0.066	2.4	Unfavor	0.47	1.22
	EPHA4	Unfavor	0.039	2.88	Unfavor	0.17	1.44
	EPHA5	Favor	0.28	0.64	Favor	0.058	0.54
	EPHA6	Favor	0.1	0.46	Favor	0.42	0.79
	EPHA7	Unfavor	0.23	1.52	Favor	0.028	0.52
	EPHA8	Unfavor	0.063	1.99	Favor	0.26	0.71
	EPHA10	Favor	0.021	0.38	Favor	0.033	0.56
	EPHB1	Favor	0.16	0.61	Unfavor	0.12	1.54
	EPHB2	Unfavor	0.0012	3.21	Unfavor	0.27	1.35
	EPHB3	Unfavor	0.27	1.5	Unfavor	0.28	1.4
	EPHB4	Unfavor	0.01	3.17	Unfavor	0.27	1.35
	EPHB6	Unfavor	0.012	2.55	Unfavor	0.039	1.87
XIV	RET	Favor	0.072	0.52	Favor	0.11	0.62
XV	RYK	Unfavor	0.012	3.06	Unfavor	0.003	2.15
XVI	DDR1	Favor	0.23	1.53	Unfavor	0.46	1.25
	DDR2	Unfavor	0.0016	3.41	Favor	0.32	0.77
XVII	ROS1	Unfavor	0.062	1.93	Favor	0.18	0.7
XVIII	AATK	Favor	0.0038	0.36	Favor	0.055	0.58
	LMTK2	Unfavor	0.23	1.79	Favor	0.0067	0.47
	LMTK3	Favor	0.21	0.6	Favor	0.093	0.64
XIX	LTK	Unfavor	0.49	1.28	Favor	0.0016	0.43
	ALK	Favor	0.0051	0.3	Favor	0.012	0.41
XX	STYK1	Unfavor	0.016	3.41	Unfavor	0.065	1.63

**Fig. 1:**
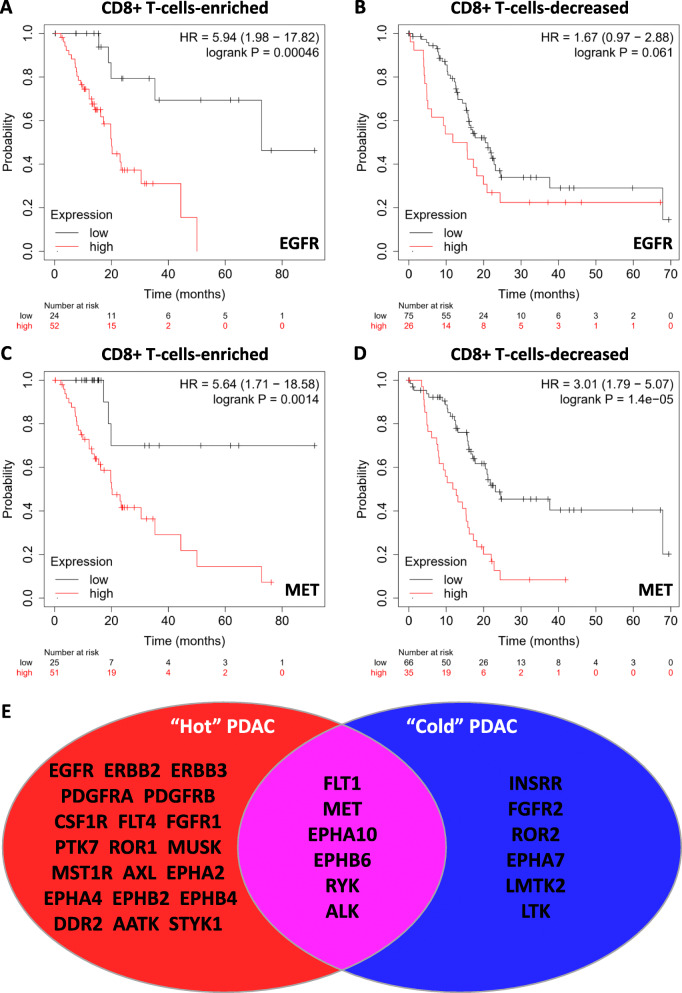
RTKs are prognostic factors for immune “hot” pancreatic cancer. **A**-**B** Overall survival analyses of patients with high and low EGFR expression in CD8 + T-cell-enriched pancreatic cancer (**A**) and CD8 + T-cell-decreased pancreatic cancer (**B**). **C**-**D** Overall survival analyses of patients with high and low MET expression in CD8 + T-cell-enriched pancreatic cancer (**C**) and CD8 + T-cell-decreased pancreatic cancer (**D**). **E** Prognostic landscape of RTKs in pancreatic cancer. All RTKs with a significant prognostic role are shown

Initially, we investigated the role of two well characterized RTKs, EGFR [43] and MET [18], in immune “hot” (CD8 + T-cell enriched) and “cold” (CD8 + T-cell decreased) pancreatic cancers. Results showed that EGFR is significantly associated with the prognosis of CD8 + T-cell-enriched pancreatic cancers and not -decreased pancreatic cancers (Fig. 1A and B). Significant prognostic association was also observed in both CD8 + T-cell-enriched and -decreased conditions in MET analyses (Fig. 1C and D). This suggests that EGFR and MET are associated with the immune response in pancreatic cancer. We subsequently expanded this analysis to all RTKs, and 32 RTKs showed a significant association with pancreatic cancer prognosis (Fig. 1E). These RTKs can be divided into six categories: (1) favorable only for CD8 + T-cell-enriched cancers (FLT4, FGFR1, and AATK); (2) favorable only for CD8 + T-cell-decreased cancers (INSRR, FGFR2, ROR2, EPHA7, LMTK2, and LTK); (3) favorable for both CD8 + T-cell-enriched and -decreased cancers (EPHA10 and ALK); (4) unfavorable only for CD8 + T-cell-enriched cancers (EGFR, ERBB2, ERBB3, PDGFRA, PDGFRB, CSF1R, PTK7, ROR1, MUSK, MST1R, AXL, EPHA2, EPHA4, EPHB2, EPHB4, DDR2, and STYK1); (5) unfavorable for both CD8 + T-cell-enriched and -decreased cancers (MET, EPHB6, and RYK); (6) unfavorable for CD8 + T cell-decreased cancers but favorable for CD8 + T-cell-enriched cancers (FLT1) (Table 1).

### RTKs are potential immunotherapeutic targets in pancreatic cancer

**Fig. 2:**
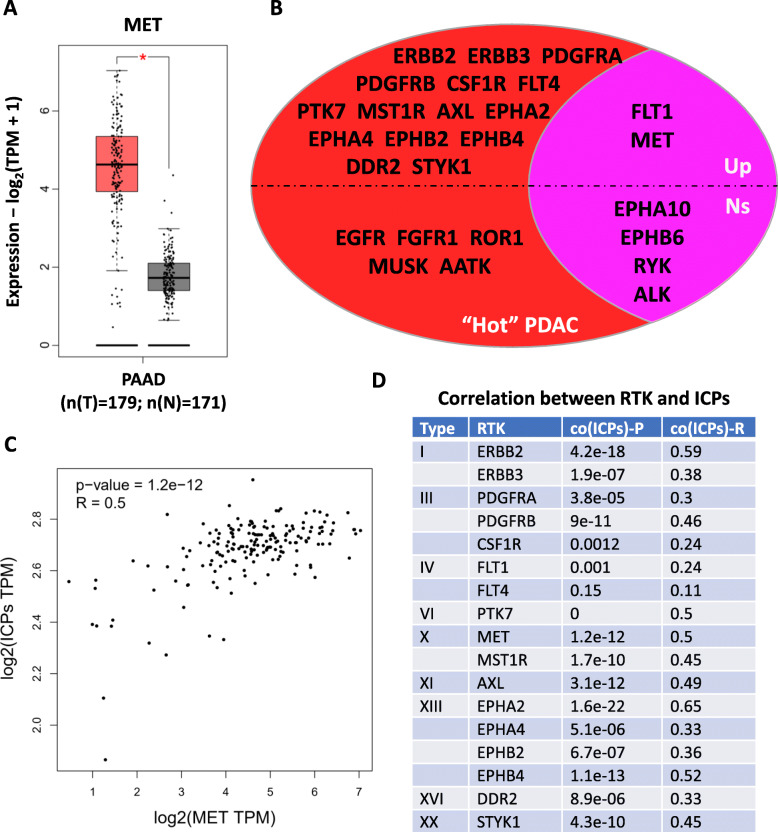
RTKs are immunotherapeutic targets in pancreatic cancer. **A** Expression analysis of MET in pancreatic cancer and normal pancreatic tissue. **B** Expression summary of RTKs. All 26 RTKs significantly associated with the prognosis of immune “hot” pancreatic cancer were subjected to differential expression analysis. Up, upregulated; Ns, not significant. **C** Correlation analysis between the expression of MET and ICPs in pancreatic cancer. **D** Correlation summary between RTKs and ICPs. All 17 RTKs significantly upregulated in immune “hot” pancreatic cancer were subjected to correlation analysis

To further investigate whether any of the 26 RTKs associated with immune “hot” pancreatic cancer are suitable immunotherapeutic targets, we compared the expression level of each RTK between pancreatic tumor tissues and normal pancreatic tissues. We found that 17 RTKs, including MET, were significantly upregulated in pancreatic cancer tissues compared with normal pancreatic tissue, suggesting that they are potentially viable therapeutic targets (Fig. 2A and B). Given that several previous studies have documented the direct and indirect regulatory effects of RTKs on ICPs, the most critical mediators for tumor immune resistance, we further analyzed the correlation between upregulated RTKs and representative ICPs (PD-L1, CD276, CD155, CD112, LGALS9, and HVEM) in pancreatic cancer and found that 16 RTKs significantly positively correlated with ICPs in terms of expression level (Fig. 2C and D). Additionally, we also investigated the correlation between each RTK and each ICP, and the results showed significantly positive associations between most of the RTKs and ICPs in pancreatic cancer. This suggests that RTKs and ICPs may synergistically contribute to immune resistance and thus should be co-targeted during pancreatic cancer treatment.

### MET is significantly associated with PD-L1 expression in pancreatic cancer

**Fig. 3:**
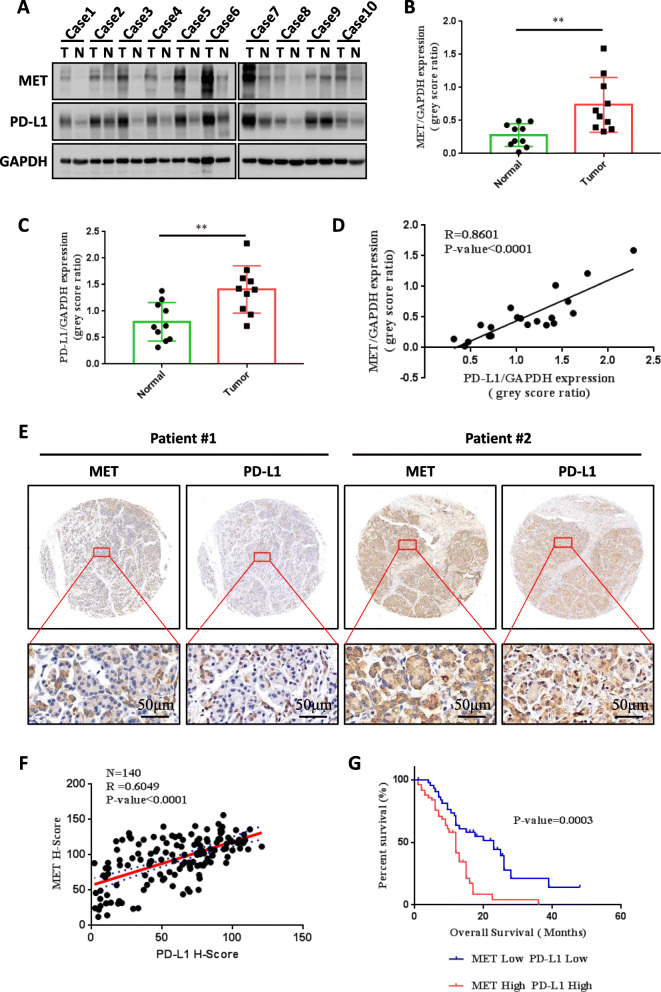
MET positively correlates with PD-L1 in PDAC patients. **A**-**C** Protein expression levels (**A**) as well as quantifications (**B** and **C**) of MET and PD-L1 in 10 paired PDAC and adjacent noncancerous tissue specimens as assessed by Western blotting (T, tumor; N, normal). **D** Quantitative correlation between MET and PD-L1 protein levels in paired PDAC and adjacent normal tissues. **E** Representative images of immunohistochemical staining for MET and PD-L1 in PDAC tissue array. Scale bar, 50 μm. **F** Quantitative correlation between MET and PD-L1 protein levels in PDAC tissue microarray. **G** Kaplan–Meier survival curves of MET–PD-L1 protein level-based OS in PDAC patients

Based on abovementioned *in silico* data, we selected MET and PD-L1 a representative ICP for further investigation. We first evaluated the expression levels of MET and PD-L1 in paired PDAC and para-cancerous tissues and found that both were markedly upregulated in PDAC tissue (Fig. 3A-C and Figures S1A-S1B). Further analysis showed a positive correlation between the expression levels of MET and PD-L1 (*p* < 0.0001, *R* = 0.8601) (Fig. 3D). We then examined MET and PD-L1 levels in tumor tissue microarray from 140 PDAC patients (Fig. 3E) and found that approximately 68 % specimens with high MET expression levels exhibited intense PD-L1 staining, confirming the positive correlation between them in pancreatic cancer (Fig. 3F). Subsequently, we assessed the associations between MET and PD-L1 expressions in terms of clinical factors and found that elevated MET and PD-L1 levels were significantly associated with lymph node invasion (*p* = 0.034), and an advanced TNM stage (*p* = 0.006) (Table 2). Moreover, patients with low MET and PD-L1 expression levels showed significantly prolonged survival compared with those with high MET and PD-L1 levels (Fig. 3G). Taken together, these data suggest that the MET–PD-L1 axis indeed functions as a prognostic marker in PDAC.

**Table 2:** Association between MET and PD-L1 expressions and clinicopathological characteristics in PDAC patients

Characteristics	MET and PD-L1	MET and PD-L1	*P* value
	low expression (*N* = 44)	high expression (*N* = 49)	
Gender			0.523
Male	28 (50 %)	28 (50 %)	
Female	16 (42.1 %)	21 (57.9 %)	
Age			0.432
≤ 60 years	14 (53.8 %)	12 (46.2 %)	
> 60 years	30 (44.8 %)	37 (55.2 %)	
BMI			0.664^#^
< 18.5	4 (40 %)	6 (60 %)	
18.5–23.9	31 (50.8 %)	30 (49.2 %)	
> 23.9	9 (40.9 %)	13 (59.1 %)	
Tumor location status			0.127
Head and neck	35 (52.2 %)	32 (47.8 %)	
Body and tail	9 (34.6 %)	17 (65.4 %)	
Tumor size status			0.501^#^
≤ 2 cm	5 (50 %)	5 (50 %)	
2 < n ≤ 4 cm	32 (50.8 %)	31 (49.2 %)	
> 4 cm	7 (35 %)	13 (65 %)	
Lymph nodes involvement			0.034
No	21 (61.8 %)	13 (38.2 %)	
Yes	23 (39 %)	36 (61 %)	
TNM stage			0.020^#^
I	15 (75 %)	5 (25 %)	
II	27 (39.7 %)	41 (60.2 %)	
III-IV	2 (40 %)	3 (60 %)	
Tumor differentiation			0.869^#^
Well differentiated	9 (60 %)	6 (40 %)	
Moderately differentiated	18 (42.9 %)	24 (57.1 %)	
Poorly differentiated	17 (47.2 %)	19 (52.8 %)	
CA199			0.575
≤ 37	7 (41.2 %)	10 (58.8 %)	
> 37	37 (48.7 %)	39 (51.3 %)	
CA125			0.597
≤ 35	33 (45.8 %)	39 (54.2 %)	
> 35	11 (52.4 %)	10 (47.6 %)	
CEA			0.196
≤ 5	30 (52.6 %)	27 (47.4 %)	
> 5	14 (38.9 %)	22 (61.1 %)	

Data are presented as numbers and percentages (in parentheses) based on the total number of patients with tumors expressing high and low MET and PD-L1 levels. *P* values were determined using Chi-square tests. #, using Fisher’s exact test; *P* < 0.05, statistically significant

## MET maintains PD-L1 expression in pancreatic cancer cells

**Fig. 4:**
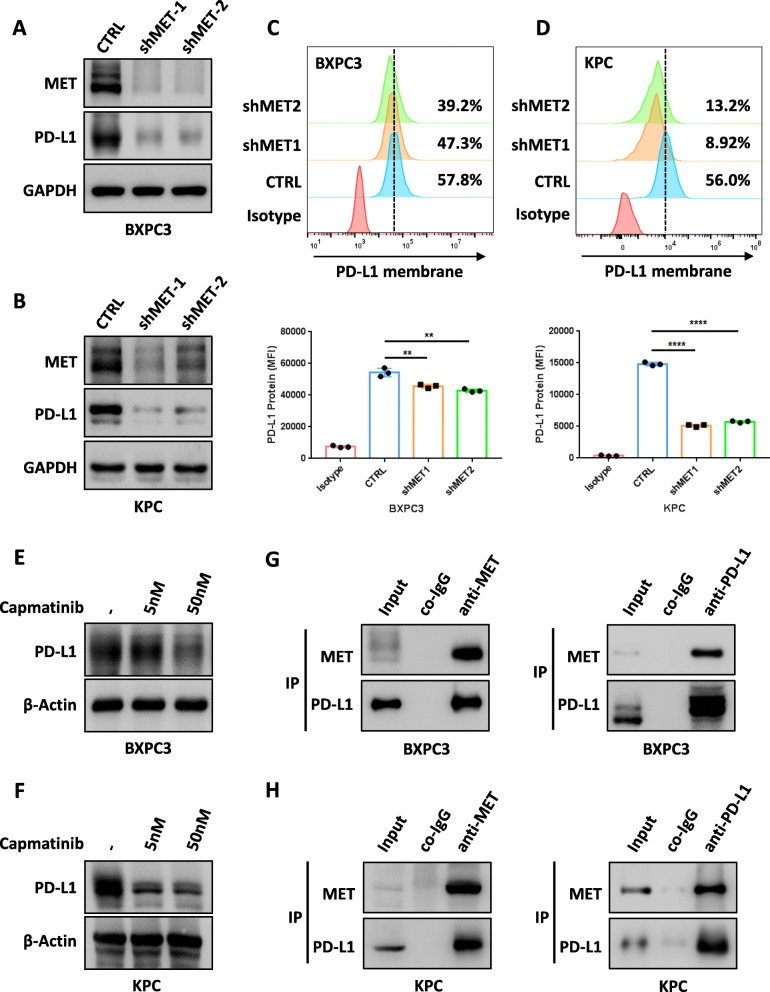
MET maintains PD-L1 expression in PDAC cells. **A** and **B** Human BXPC3 (**A**) and mouse KPC (**B**) PDAC cell lines are transfected with MET-silenced (shMET) and negative control (CTRL) vectors, respectively; MET and PD-L1 levels are assessed by immunoblotting. **C** Cell-surface PD-L1 expressions as assessed by flow cytometry in BXPC3 cells (up), with quantification (down). **D** Cell-surface PD-L1 expressions as assessed by flow cytometry in KPC cells (up), with quantification (down). **E** and **F** PD-L1 expressions as assessed by Western blotting analysis in BXPC3 (**E**) and KPC (**F**) cells after a dose-increasing treatment with capmatinib. **G** and **H** Endogenous co-immunoprecipitation of BXPC3 cells (**G**) and KPC cells (**H**) by MET and PD-L1 antibodies. MET and PD-L1 expression levels are analyzed by Western blotting

To determine the association of MET with PD-L1 in pancreatic cancer, we first investigated the influence of MET deficiency on PD-L1 expression. As excepted, PD-L1 (Fig. 4A-B) and cell-surface PD-L1 (Fig. 4C-D) expression were starkly down-regulated in the shMET group. Moreover, capmatinib, a small-molecule tyrosine kinase inhibitor (TKI) with high specificity for MET [44, 45], also induced PD-L1 downregulation in a dose-dependent manner (Fig. 4E, F). During the investigation for a potential regulatory mechanism, we found that the mRNA level of PD-L1 was remarkably reduced in the shMET group (Figure S2A). In addition, we observed decreased phosphorylation of signal transducer and activator of transcription 1 (STAT1), a critical transcriptional factor for PD-L1, and PD-L1 downregulation when MET was knocked down (Figure S2B). In support of this, IFNγ-induced phosphorylation of STAT1 and PD-L1 upregulation were inhibited in shMET group (Figure S2B, Figures S3A-S3B). Further, the administration of capmatinib significantly alleviated IFNγ-associated PD-L1 upregulation on the cell membrane (Figure S3C). Thus, MET-STAT1 axis promoted the transcription of PD-L1.

In addition to the mRNA level, the half-life of PD-L1 was shortened in the shMET group, suggesting that MET can maintain the stability of PD-L1 (Figures S4A-S4B). Further exploration showed an endogenous interaction between MET and PD-L1 (Fig. 4G, H) as well as between MET and CMTM6 (Figure S4C), a principal regulator of PD-L1. CMTM6 was found to be required for preventing PD-L1 from endo-lysosomal pathway-mediated degradation, as well as for interacting with PD-L1 to reduce its ubiquitination[46, 47]. The expression level of CMTM6 was decreased when MET was knocked down (Figure S4D). Moreover, CMTM6 overexpression abrogated the down-regulation of PD-L1 caused by MET deficiency (Figure S4E). Thus, MET maintained the stability of PD-L1 in a CMTM6-dependent manner. Taken together, MET maintained the expression of PD-L1 at least at transcriptional and post-transcriptional stages in pancreatic cancer cells.

### MET deficiency inhibits tumor growth and enhances immune cell infiltration in pancreatic cancer

**Fig. 5:**
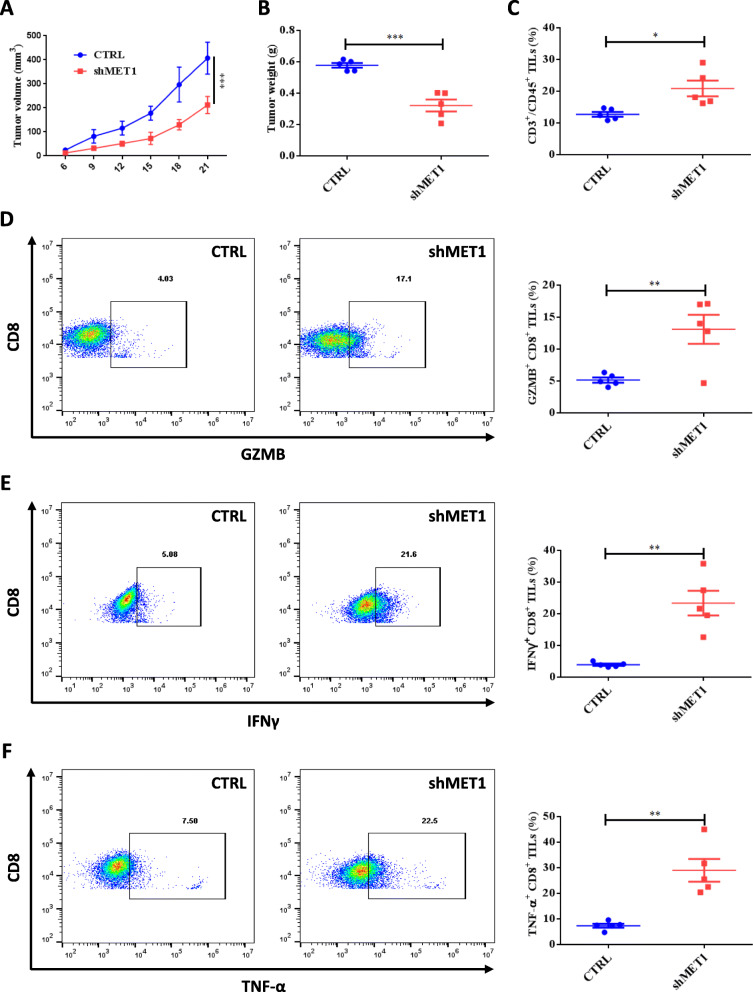
Inhibition of MET suppresses tumor PD-L1 expression and reduces tumor growth in immunocompetent mice. **A**-**B** Tumor volumes (**A**) and weights (**B**) of scrambled negative control (CTRL) and shMET-transfected KPC xenografts in C57BL/6 mice (*n* = 5). **C** Staining of CD3 + T cells among CD45 + lymphocyte populations from isolated TILs. **D** Intracellular cytokine staining of GZMB + and CD8 + cells among CD3 + T cells from purified TILs. Representative images (right) and quantification (left) are shown. **E** Intracellular cytokine staining of IFNγ + and CD8 + cells among CD3 + T-cells from purified TILs. Representative images (right) and quantification (left) are shown. **F** Intracellular cytokine staining of TNF-α + and CD8 + cells among CD3 + T cells from purified TILs. Representative images (right) and quantification (left) are shown. TILs, tumor-infiltrating lymphocytes; GZMB, Granzyme B

To determine whether MET is indeed associated with pancreatic tumor immunity, mouse PDAC KPC cells stably transfected with shMET and CTRL shRNA were individually implanted into immunocompetent C57BL/6 mice. The growth of tumors was significantly decelerated in the shMET group compared with that in the CTRL group (Fig. 5A-B). We further found that CD3 + T-cell infiltration was significantly increased in the shMET group (Fig. 5C). Similarly, the subpopulation of GZMB+, IFNγ+, and TNF-α + in infiltrated CD8 + T lymphocytes also showed significant upregulation in the shMET group (Fig. 5D, F). Taken together, these results indicate that MET silencing indeed suppressed PDAC growth in an immune-related manner.

### Dual-targeting MET and PD-L1 cooperatively inhibits pancreatic cancer

**Fig. 6:**
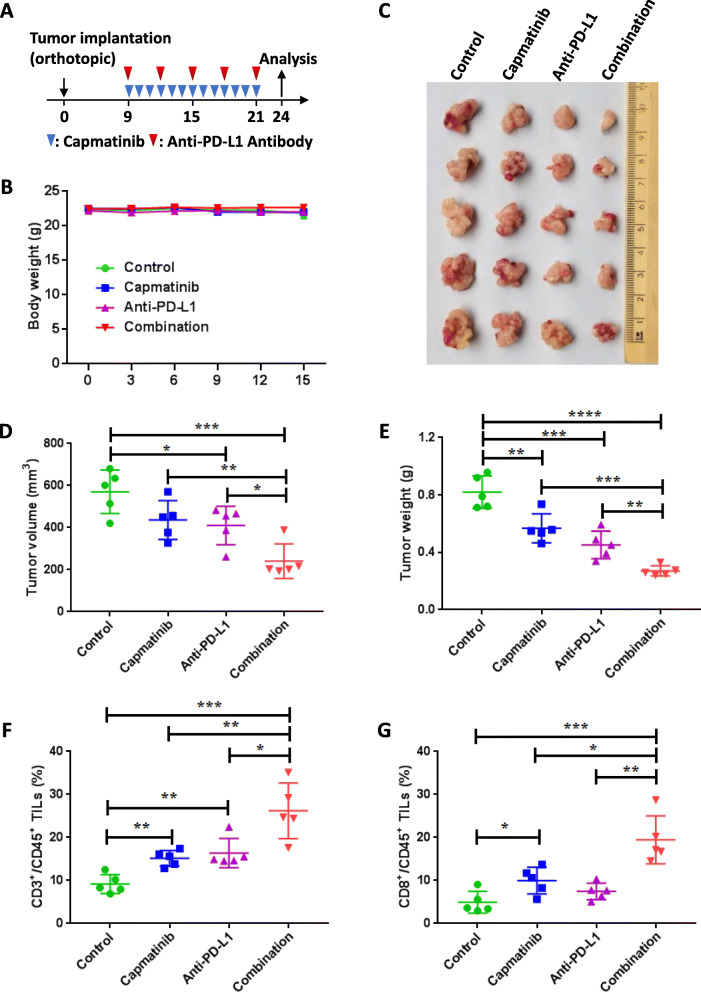
Synergistic anti-tumor effects of PD-L1 blockage and MET inhibition in an orthotopic mouse model. **A** Drug treatment protocol for capmatinib and PD-L1 antibody in C57/BL6 mice. At the endpoint, tumors were extracted and assessed via flow cytometry. **B**-**E** Sizes of orthotopic KPC xenografts after treatment with capmatinib and/or PD-L1 antibody in C57/BL6 mice. Mouse body weights (**B**), representative tumor images (**C**), tumor volumes (**D**), and tumor weights (**E**) are shown. **F** Quantification of CD3 + T cells in CD45 + lymphocytes from purified tumor-infiltrating lymphocytes. **G** Quantification of the staining of CD8 + T cells in CD45 + lymphocytes from isolated tumor-infiltrating lymphocytes

**Fig. 7:**
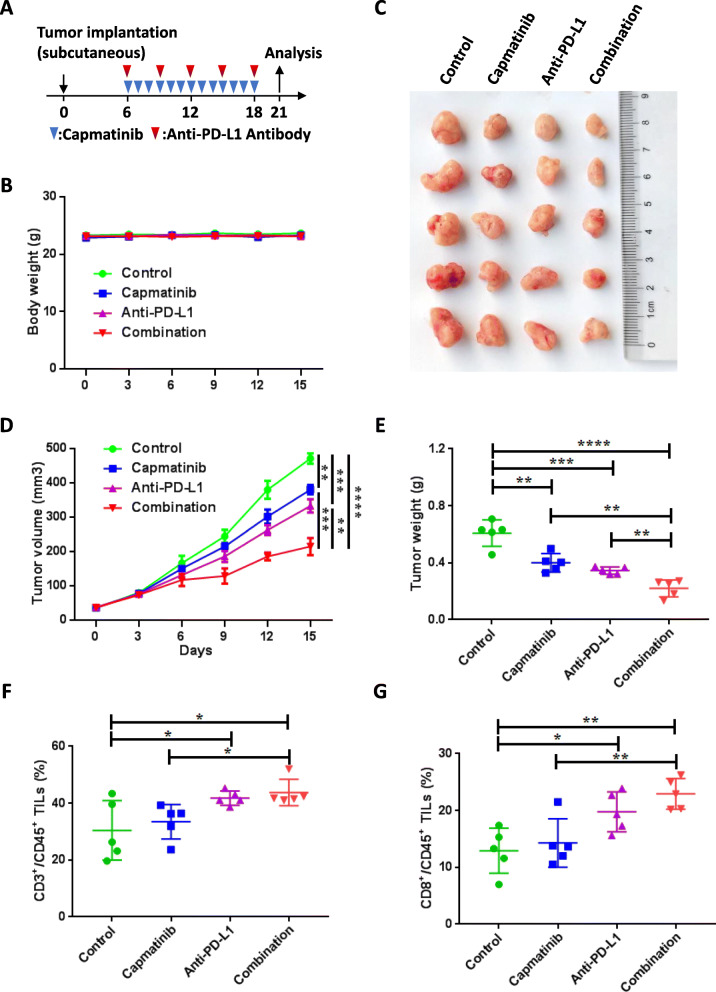
Synergistic effects of PD-L1 monoclonal antibody and MET inhibition in a subcutaneous mouse model. **A** Drug treatment protocol for capmatinib and PD-L1 antibody in C57/BL6 mice. At the endpoint, tumors were extracted and assessed via flow cytometry. **B**-**E** Sizes of subcutaneous KPC tumors in C57/BL6 mice treated with capmatinib and/or PD-L1 antibody. Mouse body weights (**B**), representative tumor images (**C**), tumor volumes (**D**), and tumor weights (**E**) are shown. **F**-**G** Quantification of CD3 + T cells (**F**) and CD8 + T cells (**G**) from purified tumor-infiltrating lymphocytes

Based on the regulatory role of MET in PD-L1 expression, we hypothesized that MET inhibition improves the therapeutic efficacy of PD-L1/PD-1 blockage. To prove this, we applied three therapeutic strategies in KPC tumor-bearing orthotopic mice, including monotherapy with capmatinib, anti-PD-L1 antibody treatment, or a combination of both (Fig. 6A). First, no significant weight loss was observed in all experimental mice (Fig. 6B). Further, as expected, the combination therapy showed more significant inhibitory effects on tumor size and burden compared with the control and both monotherapies (Fig. 6C-E). Moreover, we observed significant elevation of tumor-infiltrating CD3+, CD4+, and CD8 + T cells but not CD11B + myloid cells in mice administered combination therapy, suggesting that strategies co-blocking MET and PD-L1 synergistically potentiate anti-tumor immunity in mice (Fig. 6F-G, Figure S5A-5B). We also assessed the efficacy of combination treatment and monotherapy in a subcutaneous KPC PDAC model (Fig. 7A). Consistent with KPC-bearing orthotopic model, no significant weight change was observed after combination treatment (Fig. 7B), and significant inhibitory effects were observed on tumor size and tumor weight even in this experimental model (Fig. 7C-E). Moreover, the numbers of tumor-infiltrating CD3 + T cells and CD8 + T cells but not CD4 + T cells and CD11B + myloid cells, were also significantly elevated in mice administered with a combination of capmatinib and anti-PD-L1 antibody (Fig. 7F-G, Figures S5C-5D).

We also administered capmatinib or PD-1 antibody alone or in combination to mice bearing subcutaneous KPC tumors (Figure S7A). We found that no significant weight loss was observed in all experimental mice (Figure S6B). The combination of capmatinib and anti-PD-1 impaired tumor growth and decreased tumor burden more effectively than control treatment and capmatinib or anti-PD-1 monotreatment (Figures S6C-S6E). The levels of tumor-infiltrating CD3 + T cells and CD8 + T cells increased in mice administered capmatinib and anti-PD-1 combination (Figures S6F-S6G). Taken together, these findings indicate that MET inhibition and PD-L1/PD-1 pathway blockage cooperatively inhibited tumor growth.

## Discussion

To the best of our knowledge, this is the first study presenting a comprehensive analysis investigating an association between RTKs and pancreatic cancer prognosis, with the consideration of the immune status of patients. Moreover, in this study, we characterize the relationships between the expression levels of RTKs and ICPs in pancreatic cancer. RTKs are potential immunotherapeutic targets for immune “hot” pancreatic cancers. As shown above, the expression of different RTKs is associated with the differential prognosis of pancreatic cancer. Based on this knowledge, we believe that the treatment strategy for each patient should be individualized via precision medicine. Specifically, we identified three RTKs, MET, EPHB6, and RYK, which are associated with a poor prognosis regardless of the immune status in patients with pancreatic cancer. We suggest that further preclinical validation of the inhibitory potential of these RTKs be conducted using blockage antibodies or small molecule inhibitors. In contrast, we identified two RTKs, EPHA10 and ALK, which are associated with a good prognosis in patients with pancreatic cancer regardless of the immune status. For these RTKs, we suggest that strategies to activate them, rather than inhibit them, using natural ligands may be therapeutically beneficial. Of note, we also identified RTKs (e.g., FLT1, also known as VEGFR1) showing contrasting effects on prognoses between immune “hot” and “cold” cancers. For these RTKs, we suggest that the infiltration of CD8 + T cells be detected before deciding how to manage these patients.

Recently, Li et al. have reported that MET suppression upregulates PD-L1 in liver cancer, and MET knockout (KO) induces PD-L1 expression by preventing GSK3B-associated PD-L1 degradation rather than by affecting its transcription [48]. In contrast, our report first revealed that high MET expression has a positive correlation with PD-L1 upregulation in PDAC. This is consistent with another previous study reporting that elevated PD-L1 expressions are correlated with high MET levels in metastatic clear cell renal cell carcinoma [49]. Interestingly, we showed that MET deficiency resulted in reduced protein stability of PD-L1, thereby lowering PD-L1 expression in pancreatic cancer cells. Burr et al.[47] identified CMTM6 as a critical regulator of PD-L1 in a broad range of cancer cells, and reported that CMTM6 is required for efficient endocytic recycling of PD-L1, thus preventing PD-L1 degradation in lysosomes. Mezzadra et al. [46] demonstrated that CMTM6 associates with the PD-L1 protein, reducing its ubiquitination and increasing PD-L1 protein half-life. In this study, MET deficiency-induced PD-L1 downregulation could be restored by overexpressing CMTM6. In addition to decreased stability, MET deficiency could induce mRNA level downregulation of PD-L1, which might be associated with the inactivation of STAT1. These results imply that MET regulates PD-L1 expression probably at the transcriptional, translational, and post-translational levels. Additionally, it is well-established that IFNγ upregulates PD-L1 expression to promote cancer immune resistance [50, 51]. Previous studies have mainly focused on IFNγ-induced activation of IRF1, which directly interacts with the PD-L1 promoter to enhance its transcription via the JAK-STAT signaling pathway [52]. A recent study reported that IFNγ-associated PD-L1 upregulation in medulloblastoma is mediated by CDK5, the depletion of which downregulates PD-L1 in malignant cells [53]. In this study, we revealed that MET may be a crucial mediator of IFNγ-associated PD-L1 upregulation in PDAC. Taken together, these results suggest that MET-mediated PD-L1 regulation likely involves several proteins and has different activation mechanisms in different cancers.

To date, anti-PD-1/PD-L1 administration has been regarded as one of the most successful immunotherapeutic options in various tumors [54]. However, its overall response rate usually does not exceed 40 % across multiple cancer types because relevant regulatory mechanisms of PD-L1 expression potentially influence the efficacy of ani-PD-L1 therapy [55]. Increasing evidence reveals that co-targeting these regulatory pathways with PD-L1 can improve the efficacy of immunotherapy in patients [56, 57]. Capmatinib (INC280), an efficient and specific MET suppressor, is a promising anti-tumor drug [58]. This was proved in a phase II study in individuals with advanced liver cancer who expressed high levels of MET [59]. In the current study, tumor growth was inhibited in the mono-capmatinib group compared with the controls. Moreover, capmatinib enhanced the efficacy of anti-PD-L1 in mouse models, indicating the potential benefits of a combined therapeutic strategy for treating pancreatic cancer. However, the vast majority of PDAC patients are diagnosed at advanced or metastasized stages. Compared with this, the treatment for mice starts early, which is discrepant with the real-word patient situation. Therefore, further studies targeting RTKs are warranted in the future for the clinical application of our findings.

## Conclusions

In conclusion, this study showed that dual blocking of MET and PD-L1 enhances pancreatic cancer immunotherapy. Despite the conflicting correlation between RTKs and ICPs in other cancer types [26, 48, 60, 61], we have taken critical first steps to improve therapeutic options in pancreatic cancer. Anomalous expression of RTKs may be used as a prognostic factor to design tailored therapeutic strategies for individual cancer patient. However, to provide immediate clinical significance, this work will need to be extended to clinical studies to validate the strategy of RTK targeting in pancreatic cancer in combination with ICP therapy, as appropriate.

## Supplementary Information

**Additional file 1: Figure S1.** MET overexpression in PDAC tissue samples. (A-B) MET protein expressions in 18 paired PDAC and adjacent noncancerous tissue specimens assessed immunohistochemically. T, tumor; N, normal. **Figure S2.** MET promotes transcriptional upregulation of PD-L1 in PDAC cells. (A) MET (up) and PD-L1 (down) mRNA expressions in BXPC3 cells. The cells were transfected with shMET or CTRL shRNA as assessed by qPCR. (B) MET, STAT1, pSTAT1 and PDL1 protein levels are assessed by immunoblotting in BXPC3 and KPC cells were transfected with MET silenced (shMET) and/or were treated with IFNγ. **Figure S3.** MET inhibition hampers IFNγ-induced PD-L1 upregulation in PDAC cells. (A) BXPC3 cells underwent transfection with shMET or CTRL shRNA, and were treated with IFNγ for 48 h, followed by PD-L1 level assessment by flow cytometry. Representative images (right) and quantification (left) are shown. (B) KPC cells underwent transfection with shMET or CTRL shRNA, and were treated with IFNγ for 48 h, followed by PD-L1 level assessment by flow cytometry. Representative images (right) and quantification (left) are shown. (C) PD-L1 cell membrane protein expression in KPC cells after 48 h of treatment with IFNγ alone or IFNγ combined with capmatinib. Representative flow-cytograms (right) and quantification (left) are shown. **Figure S4.** MET inhibits protein degradation of PD-L1 in PDAC cells. (A-B) Vector control and shMET BXPC3 (A) and KPC cells (B) were treated with CHX (100 µg/mL) and analyzed by Western blotting to determine the stability of PD-L1 protein. (C) Endogenous co-immunoprecipitation of BXPC3 cells by MET and CMTM6 antibodies. MET and CMTM6 expression levels are analyzed by Western blotting. (D) MET and CMTM6 protein levels are assessed by immunoblotting in BXPC3 cells transfected with MET silenced (shMET) or negative control (CTRL) vectors. (E) MET, CMTM6, and PD-L1 protein levels are assessed by immunoblotting in BXPC3 cells transfected with MET silenced (shMET) and/or CMTM6 overexpressed (CMTM6-OE). **Figure S5.** Immuno-profiling of combinational blockade of MET and PD-L1 in orthotopic and subcutaneous mouse models. (A-B) Quantification of CD4+ T cells from purified tumor-infiltrating lymphocytes in an orthotopic (A) and a subcutaneous (B) mouse model. (C-D) The myeloid cell density in tumors was quantitated based on the area of positive IHC staining of the CD11B marker in an orthotopic (C) and a subcutaneous (D) mouse model. **Figure S6.** Synergistic effects of PD-1 monoclonal antibody and MET inhibition in a subcutaneous mouse model. (A) Drug treatment protocol for capmatinib and PD-1 antibody in C57/BL6 mice. At the endpoint, tumors were extracted and assessed via flow cytometry. (B-E) Sizes of subcutaneous KPC tumors in C57/BL6 mice treated with capmatinib and/or PD-1 antibody. Mouse body weights (B), representative tumor images (C), tumor volumes (D), and tumor weights (E) are shown. (F-G) Quantification of CD3+ T cells (F) and CD8+ T cells (G) from purified tumor-infiltrating lymphocytes.

## Data Availability

All datasets analyzed in the current study are available in the TCGA database. All data generated during this study are included in the article. Further discussion for this study and potential collaboration will be available from the corresponding authors upon reasonable request.

## Abbreviations

RTK: Receptor tyrosine kinase
TKI: Tyrosine kinase inhibitor
PDAC: Pancreatic ductal adenocarcinoma
EGFR: Epidermal growth factor receptor
HGF: Hepatocyte growth factor
ICP: Immune checkpoint
ICB: Immune checkpoint blockage
PD-1: Programmed cell death protein 1
PD-L1: PD-1 ligand 1
TCGA: The Cancer Genome Atlas
GEPIA: Gene Expression Profiling Interactive Analysis
STAT1: Signal transducer and activator of transcription 1
GZMB: Granzyme B
