# Supplementary figures and images for: Combinational blockade of MET and PD-L1 improves pancreatic cancer immunotherapeutic efficacy
Source: J Exp Clin Cancer Res. 2021 Sep 3;40:279. doi: 10.1186/s13046-021-02055-w (PMC8414725; doi:10.1186/s13046-021-02055-w)

**A**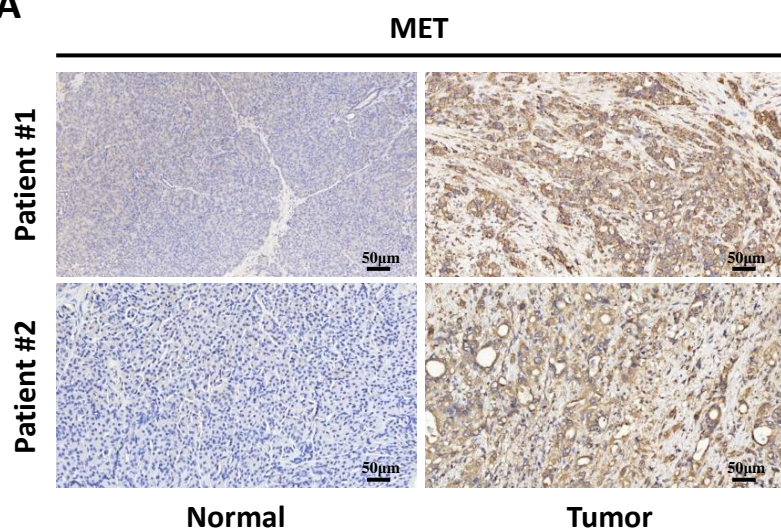**B**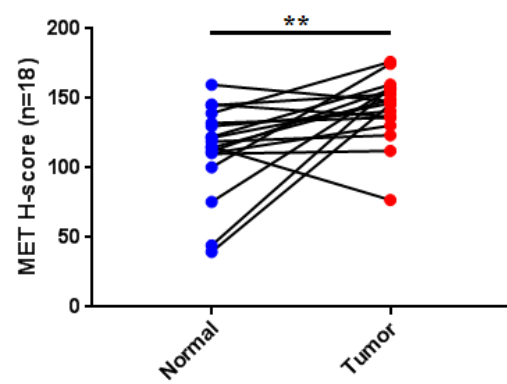**Figure S1**

**A**

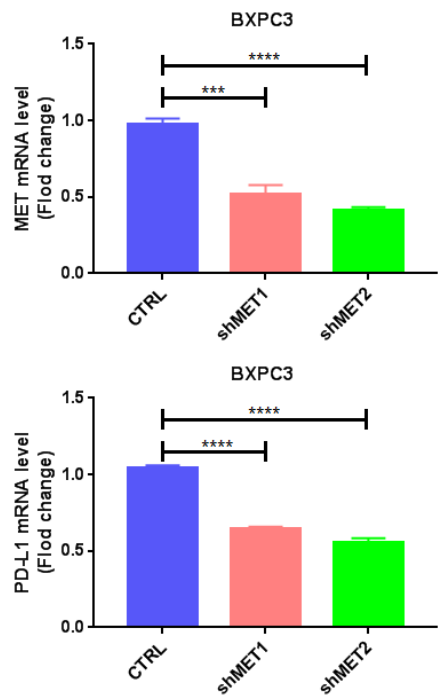

**B**

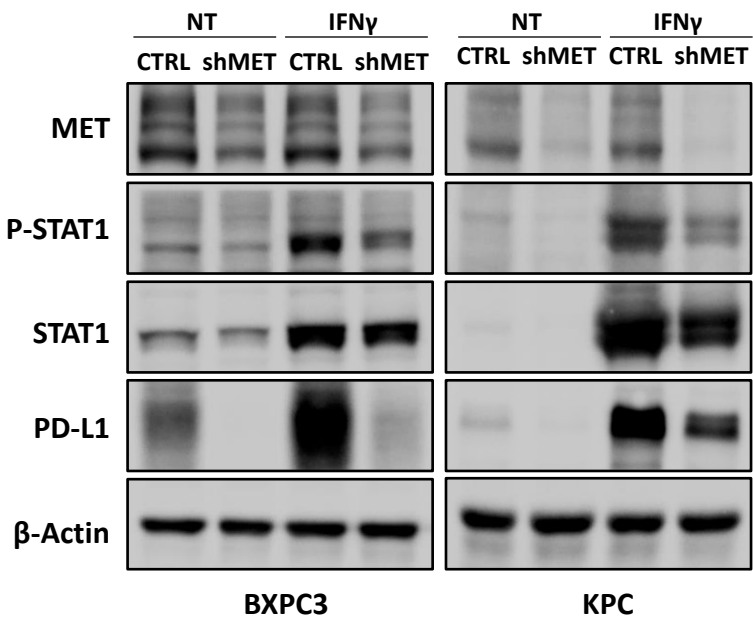

**Figure S2**

**A**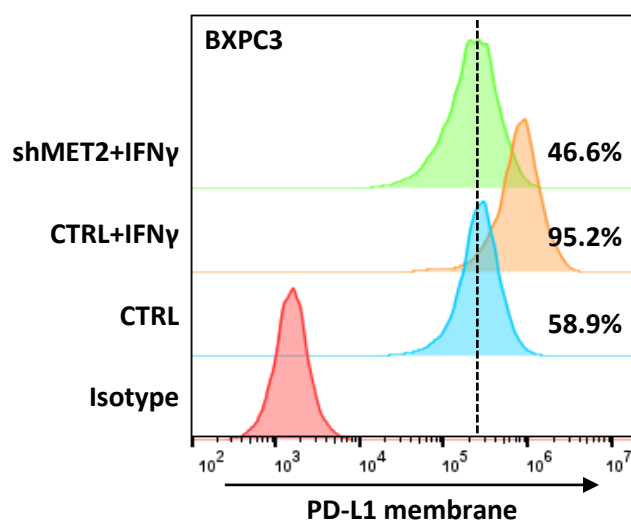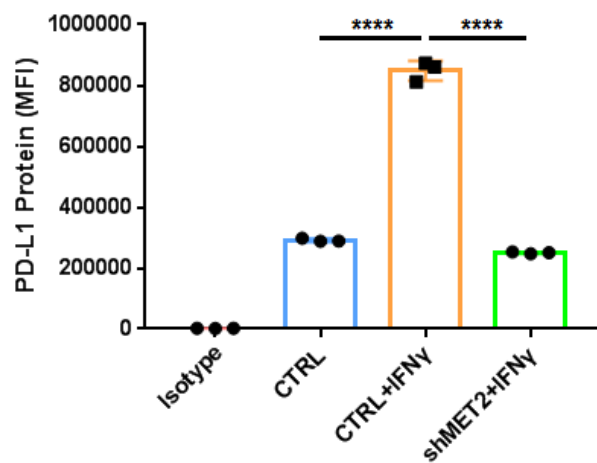**B**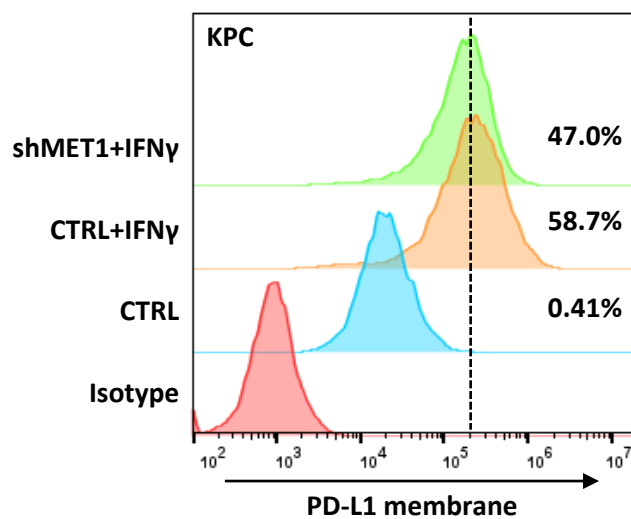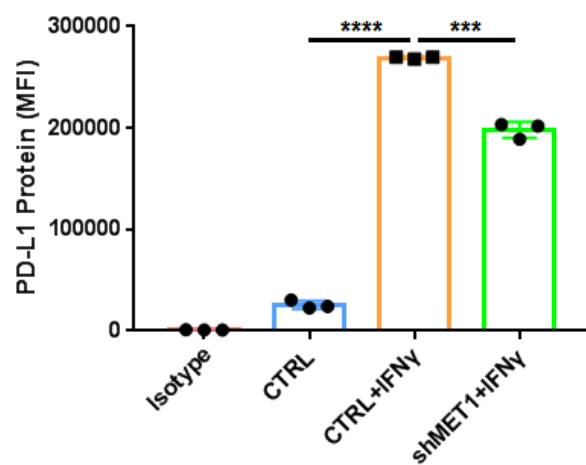**C**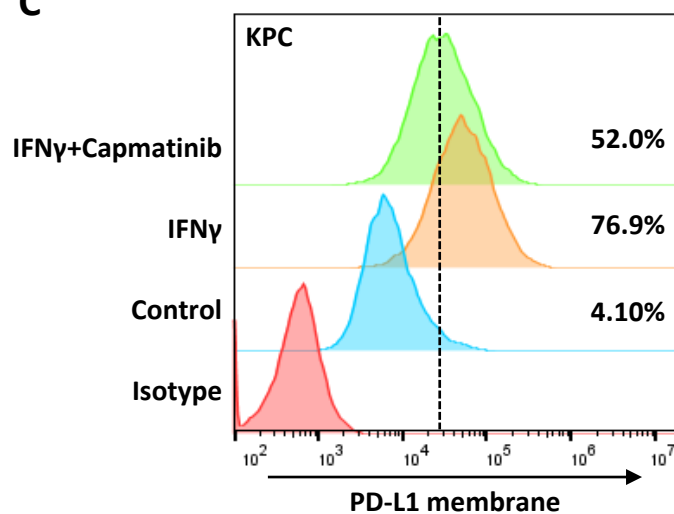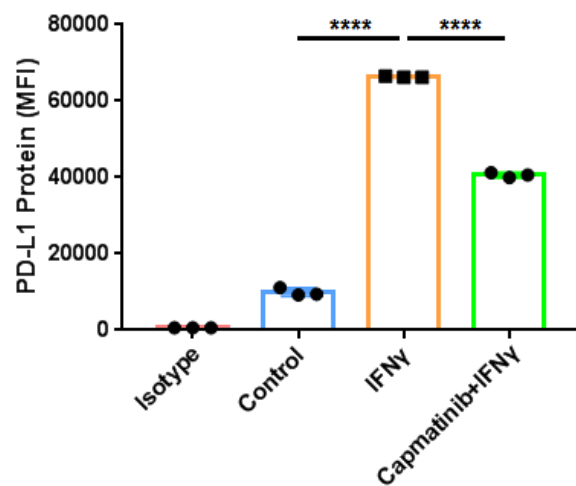**Figure S3**

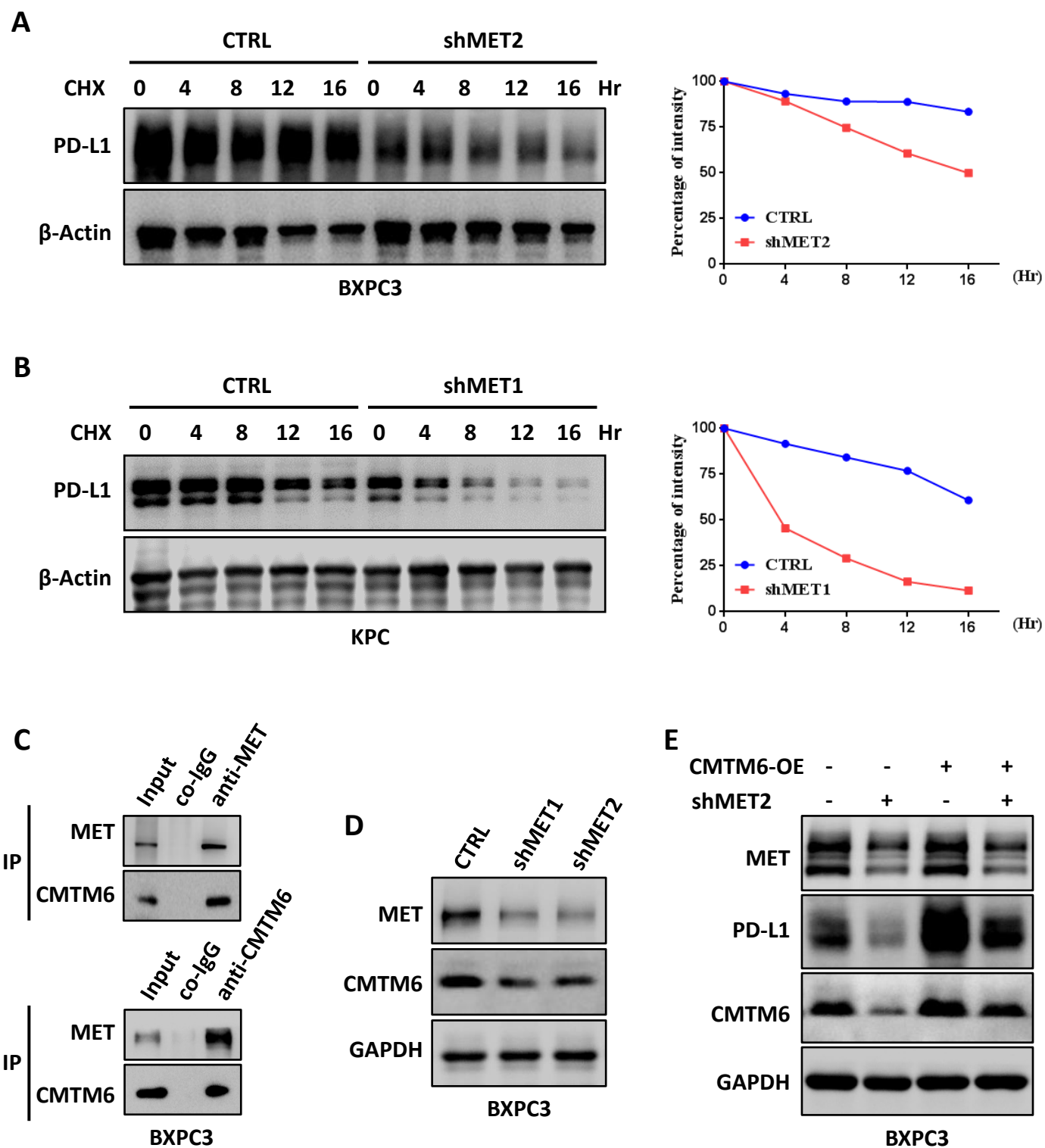

Figure S4

**A**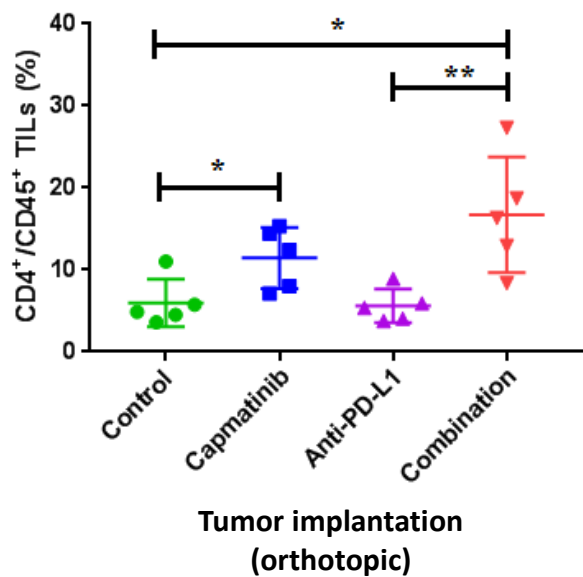**C**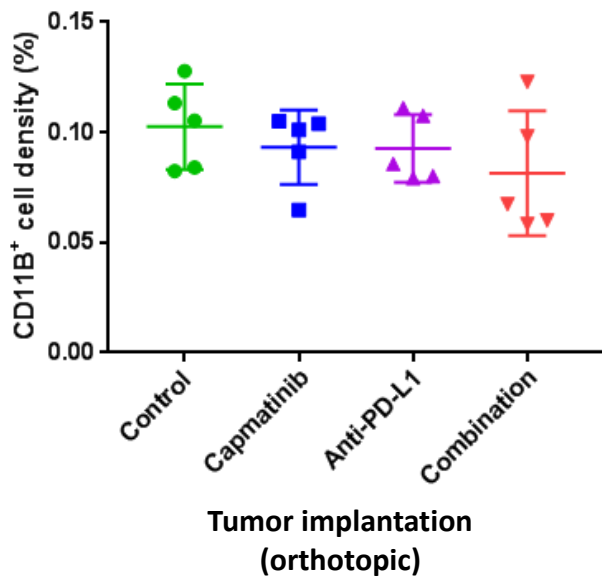**B**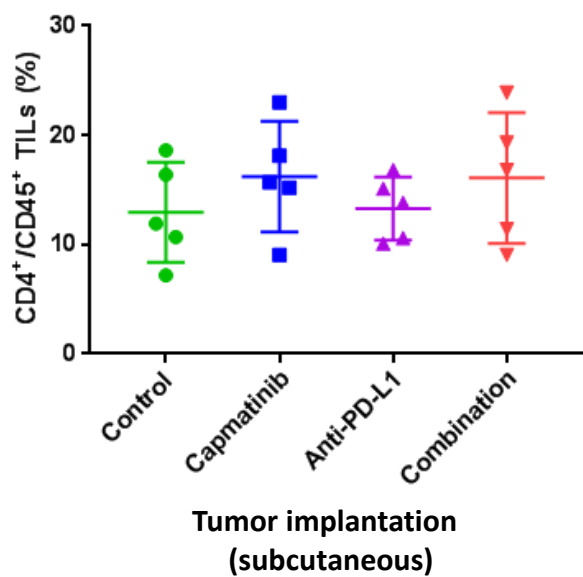**D**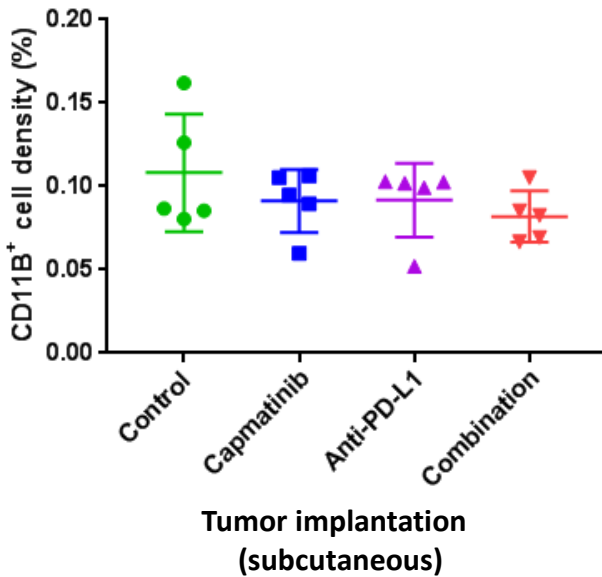**Figure S5**

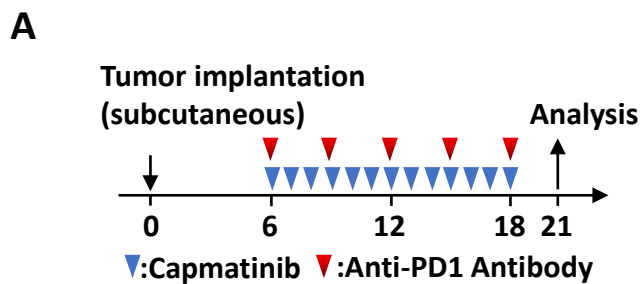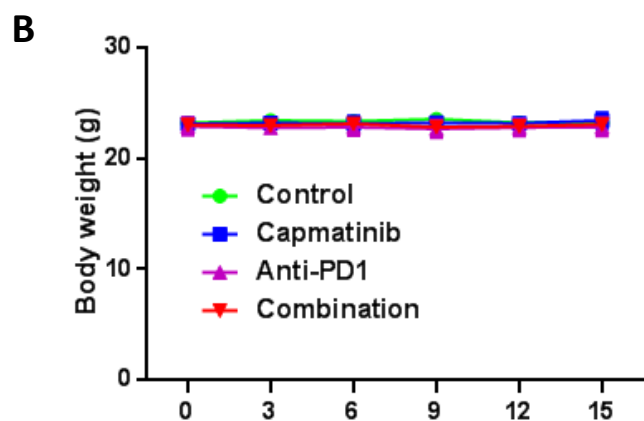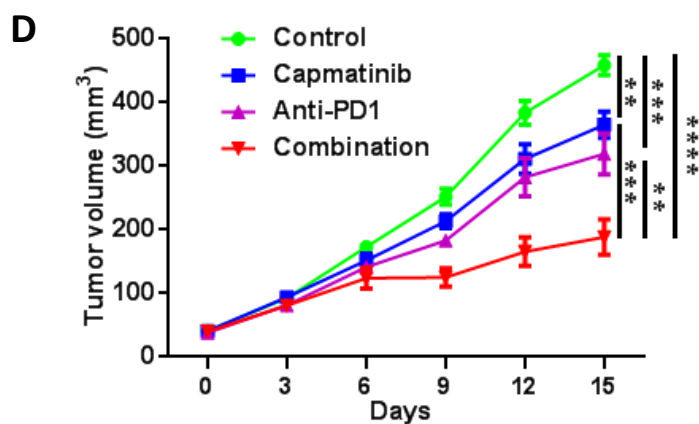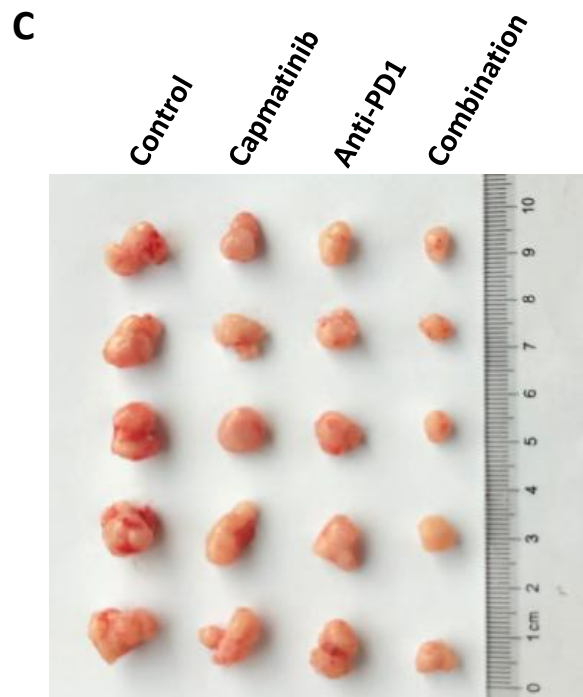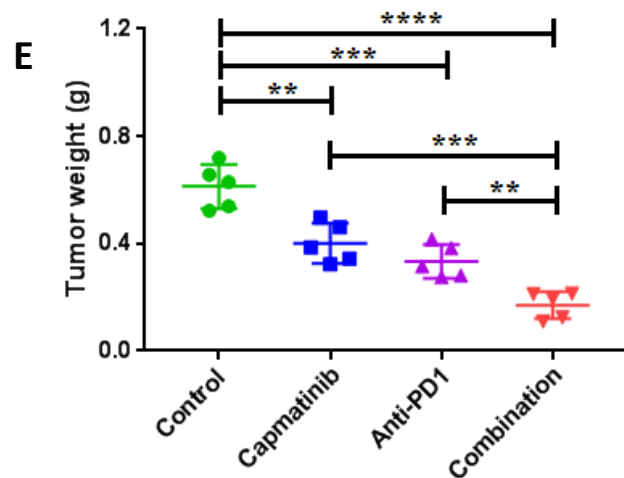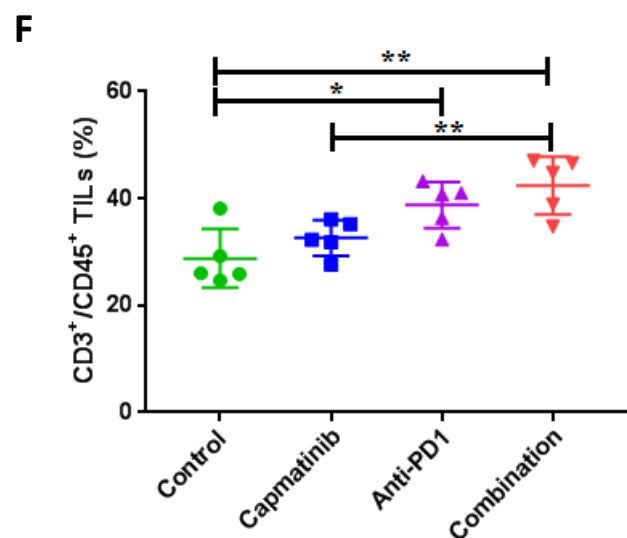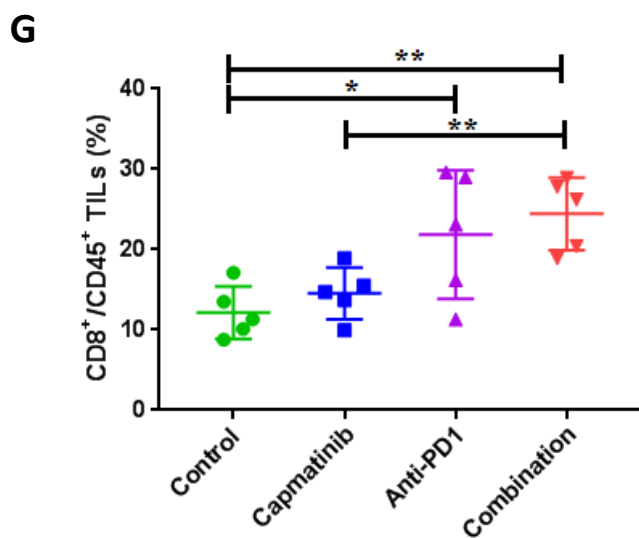

Figure S6

Supplement: Supplementary file 1 — Additional file 1: Figure S1. MET overexpression in PDAC tissue samples. (A-B) MET protein expressions in 18 paired PDAC and adjacent noncancerous tissue specimens assessed immunohistochemically. T, tumor; N, normal. Figure S2. MET promotes transcriptional upregulation of PD-L1 in PDAC cells. (A) MET (up) and PD-L1 (down) mRNA expressions in BXPC3 cells. The cells were transfected with shMET or CTRL shRNA as assessed by qPCR. (B) MET, STAT1, pSTAT1 and PDL1 protein levels are assessed by immunoblotting in BXPC3 and KPC cells were transfected with MET silenced (shMET) and/or were treated with IFNγ. Figure S3. MET inhibition hampers IFNγ-induced PD-L1 upregulation in PDAC cells. (A) BXPC3 cells underwent transfection with shMET or CTRL shRNA, and were treated with IFNγ for 48 h, followed by PD-L1 level assessment by flow cytometry. Representative images (right) and quantification (left) are shown. (B) KPC cells underwent transfection with shMET or CTRL shRNA, and were treated with IFNγ for 48 h, followed by PD-L1 level assessment by flow cytometry. Representative images (right) and quantification (left) are shown. (C) PD-L1 cell membrane protein expression in KPC cells after 48 h of treatment with IFNγ alone or IFNγ combined with capmatinib. Representative flow-cytograms (right) and quantification (left) are shown. Figure S4. MET inhibits protein degradation of PD-L1 in PDAC cells. (A-B) Vector control and shMET BXPC3 (A) and KPC cells (B) were treated with CHX (100 µg/mL) and analyzed by Western blotting to determine the stability of PD-L1 protein. (C) Endogenous co-immunoprecipitation of BXPC3 cells by MET and CMTM6 antibodies. MET and CMTM6 expression levels are analyzed by Western blotting. (D) MET and CMTM6 protein levels are assessed by immunoblotting in BXPC3 cells transfected with MET silenced (shMET) or negative control (CTRL) vectors. (E) MET, CMTM6, and PD-L1 protein levels are assessed by immunoblotting in BXPC3 cells transfected with MET silenced (shMET) [file 13046_2021_2055_MOESM1_ESM.pdf]
